# Iris Ring Melanoma Presenting as Scleral Pigmentation

**DOI:** 10.1155/2022/4840380

**Published:** 2022-07-04

**Authors:** Matej Zupan, Alenka Lavrič, Jože Pižem, Katrina Novak Andrejčič

**Affiliations:** ^1^Eye Hospital, University Medical Centre Ljubljana, Ljubljana, Slovenia; ^2^Institute of Pathology, Medical Faculty University of Ljubljana, Ljubljana, Slovenia

## Abstract

To report a patient with a very rare variant of iris melanoma that grows in the shape of a ring (ring melanoma). A 65-year-old patient was examined because of a pigmented lesion on the sclera. After a complete ophthalmic and ultrasound examination, a ring melanoma was diagnosed. Enucleation of the affected eye was performed, and histology report confirmed iris ring melanoma. This type of malignancy represents an exceedingly rare variant of uveal melanoma, and because of atypical clinical picture, it can be easily overlooked or misdiagnosed, which often delays adequate treatment. Gonioscopy, transillumination, and ultrasound help us to recognize and diagnose ring melanoma. Suspicion should be raised with a clinical picture that shows unilateral pigmentary glaucoma. The objective of this presentation is to describe and outline the challenging diagnosis and management of this rare disease entity.

## 1. Introduction

Ring melanoma can involve the choroid, the ciliary body, the anterior chamber angle, and the iris. The first ring melanoma of the iris and the ciliary body was described by Ewetzky [1]. It is an uncommon and frequently misdiagnosed variant of uveal melanoma, typically known for its circumferential growth around the eye. In most cases, the structures of the anterior chamber angle and the outflow canals get invaded, which leads to secondary glaucoma. Increased intraocular pressure is therefore a common first sign of a tumor, posing a challenge to ophthalmologists in arriving at the appropriate diagnosis.

We present the case of an exceedingly rare variant of iris melanoma that grows in the shape of a ring.

## 2. Case Presentation

**Figure 1:**
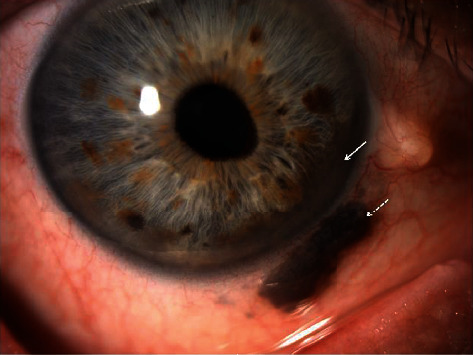
A slit-lamp photo revealing a pigmented iris mass (white arrow) with an extrascleral spread (dashed arrow).

**Figure 2:**
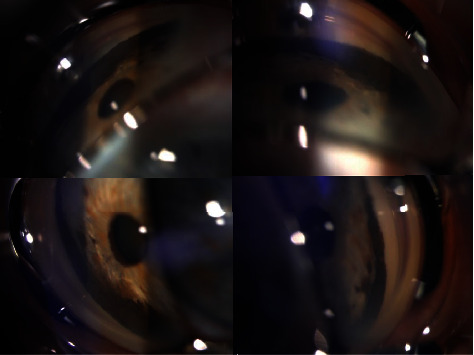
Gonioscopy showed a circumferential pigmented angle infiltration.

**Figure 3:**
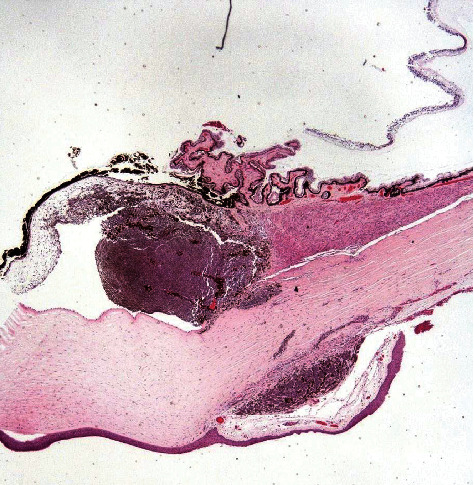
Microscopical examination demonstrating iris melanoma with extension in the anterior angle, infiltration of the trabecular meshwork and extrascleral extension (black arrow) (hematoxylin and eosin, original magnification, 40×).

A 65-year-old male without significant medical history presented with a pigmented lesion in the sclera of the right eye, accidentally noticed by his relatives. He was asymptomatic in both eyes. His best-corrected visual acuities were 20/20 in both eyes. Intraocular pressures were 35 and 15 mmHg, respectively. The results of the left eye examination including gonioscopy and ultrasound were completely normal. The ophthalmic examination of the right eye revealed a darkly pigmented extrascleral lesion in the lower nasal quadrant (Figure 1). The cornea was clear without any anterior chamber inflammation. There was a pigmented elevated iris mass from 3 to 6 o'clock and mild corectopia. The gonioscopy revealed a pigmented thickening of the iris base in the lower nasal quadrant and a 300° pigmented angle infiltration (Figure 2). The ultrasound biomicroscopy (UBM) showed no ciliary body lesions but confirmed that the iris base was thickened inferonasally. The results of the dilated right fundus examination were normal. After the ophthalmic and ultrasound examinations, ring melanoma was diagnosed. The patient additionally underwent an abdomen ultrasound and a chest X-ray exam, which showed no distant metastasis. Enucleation of the affected eye was performed. The histology report confirmed iris ring melanoma, which was growing in the root area of the iris, on the surface of the anterior chamber angle, and in the trabecular meshwork in a nearly closed circle, from 10 to 8 o'clock (Figure 3). Melanoma was mixed cell type with mitotic rate 2/1mm^2^, moderate melanin pigment, and mild intratumoral lymphocytic infiltration. Our patient continues to undergo annual systemic surveillance with hepatic ultrasound. Three years after the diagnosis, he showed no evidence of systemic metastatic disease.

## 3. Discussion

Iris melanoma accounts for around 3% of all uveal melanomas, whereas ring melanoma of the anterior chamber angle, the iris, or the ciliary body represents even fewer uveal melanomas, approximately 0.2 to 0.3% [2–4].

Due to the rarity of ring melanoma, a correct diagnosis is usually delayed, leading to poor prognosis and high metastasis rate. In the largest series of 14 cases of ring melanoma of the anterior chamber angle, an intraocular malignancy was discovered at a mean of 8 months after presentation [3]. Patients are most commonly misdiagnosed with pigmentary glaucoma, which is typically bilateral. It is therefore very important to raise suspicion when the clinical picture of elevated unilateral intraocular pressure persists and reevaluates the primary diagnosis, especially when hyperpigmentation in the anterior part of the eye is present. Furthermore, we should pay attention to other common signs and symptoms of pigmentary glaucoma, usually bilateral disease, which often presents itself in young miopic individuals with deep anterior chamber, Krukenberg spindle, dense trabecular meshwork pigmentation, and midperipheral iris transillumination defects [3–5]. This is crucial because early diagnosis and adequate treatment are of high significance in preventing distant metastasis. Invasive open intraocular surgical or laser procedures, such as laser peripheral iridotomy, should be avoided if the presence of a tumor is suspected to prevent the spread of tumor cells [6].

In the presented case, the first sign was an extrascleral pigmented mass, and similar iris ring melanoma with extrascleral extension has been previously reported in the literature [7]. Acute angle closure with reduced vision and severe pain can also appear as a presenting sign [6]. Gonioscopy, UBM, and anterior segment optical coherence tomography (AS-OCT) are crucial for evaluating the anterior angle structure and for promptly diagnosing ring melanoma [3]. UBM has the best ability to penetrate through larger tumors, especially when examining highly pigmented lesions and ciliary body tumors [8]. These findings are in line with the report from Bianciotto et al. confirming that UBM is superior to AS-OCT in visualizing the entire tumor configuration and posterior tumor margin [9]. On the other hand, the noncontact AS-OCT technique is reliable in small nonpigmented anterior iris lesions [10, 11]. Transillumination showed blockage of light in all patients in a series of cases of ring melanoma of the ciliary body, and it can be helpful in patients with opaque media, but may be nonrevealing in ring melanoma of angle structure [3, 4]. A fine needle aspiration biopsy (FNAB) of the iris or the ciliary body mass is advised in difficult cases such as an amelanotic ring melanoma [12, 13]. Char et al. reported 69% of true positive FNAB results in 16 cases of iris ring melanoma, stressing that the major limitation of their technique was the paucicellular specimens for cytopathological analysis [12]. Possible treatment options for ring melanoma of the anterior chamber angle and the ciliary body rarely include plaque and proton radiotherapy, and in most cases, enucleation is advised [3, 4, 14]. Demirci et al. reported that 52% of ring melanoma patients developed systemic metastasis after a mean follow-up of 55 months [4].

The anterior angle or iris root involvement is a poor prognostic sign in iris melanoma. It was proposed that angle involvement and high ocular pressure may allow the tumor to spread to the Schlemm canal and pose the risk of possible hematogenous expansion [2, 3]. Although life prognosis in ring melanoma of the angle structure or the ciliary body remains guarded, by the time of the three-year follow-up, our patient did not develop any metastases and continues with six-month systemic surveillance.

## Data Availability

The data that support the findings of this study are available from the corresponding author upon reasonable request.
